# Laparoscopic repair of wound defects in the elderly: our experience of 5 years

**DOI:** 10.1186/1471-2482-13-S2-S23

**Published:** 2013-10-08

**Authors:** Alessia G Ferrarese, Valter Martino, Stefano Enrico, Alessandro Falcone, Silvia Catalano, Enrico Gibin, Silvia Marola, Alessandra Surace, Mario Solej

**Affiliations:** 1 University of Turin, Department of Oncology, School of Medicine, Teaching Hospital "San Luigi Gonzaga", Section of General Surgery, Orbassano, Turin, Italy

## Abstract

**Background:**

Laparoscopic approach for wound defects is a procedure that aims to reduce surgical aggressiveness against the abdominal wall by using minimal incisions and dedicated instruments.

**Methods:**

We report our experience about clinical outcome of elderly patients undergoing laparoscopic repair for incisional hernias (Group I) and primary inguinal hernias (Group II) from June 2007 to September 2012.

We analyzed preoperative and postoperative data for the laparoscopic approach in the elderly.

**Results and discussion:**

In our experience there was no significant difference in laparoscopic procedure between normalweight and overweight patients.

**Conclusions:**

Laparoscopic repair for primary inguinal hernias and incisional ventral hernias with transabdominal placement of composite mesh in the elderly achieves excellent results with lower morbidity in comparison with open surgical approaches.

## Background

Laparoscopic approach of wound defects is a procedure that aims to reduce surgical aggressiveness against the abdominal wall by using minimal incisions and dedicated instruments. The procedure also aims to decrease total hospital stay and morbility [1-3].

In the literature there are few study about wound defects in elderly [4-9].

We report our experience about clinical outcome of elderly patients undergoing laparoscopic repair for incisional hernias (Group I) and primary inguinal hernias (Group II). Aim of this study is to assess the safety of laparoscopic wound defects repair in elderly patients; moreover the study wants to test our technique efficacy after 5 years of experience.

## Methods

From June 2007 to September 2012 we performed 63 laparoscopic wound defects repair in 52 elderly patients. Exclusion criteria were: anesthesiologic contraindication for laparoscopy, ASA IV score. We didn't exclude ASA III patients: in these patients we used abdominal pressure not superior to 10 mmHg ^(1 VLC)^. The sample was composed by 37 male and 15 female patients. We performed 21 surgical incisional hernia repair and 42 primary hernia repair. Of those, 17 were for incisional single defect hernias (24.5% recurrence hernias), 4 for multiple defects (7,54% recurrence hernias); 18 laparoscopic repairs were performer for unilateral inguinal hernias (22,56% recurrence hernias), 16 for bilateral inguinal hernias (30,08% recurrence hernias), 4 for umbilical hernias (1 recurrence hernias), 2 for epigastric and linea alba's hernias, and 2 for rectum diastasis.

Wound defects diagnosis based on objective and clinical aspects, physical examination and radiologic findings like US and CT. For this study we also considered: hernia defect size, recurrence rate and operative time. All procedure-related complications were evaluated. 42% of the patients were normalweight with a mean BMI of 25 kg/m2, 41% 25 > BMI > 30 (overweight), 17% BMI > 30 (obesity).

## Results and discussion

There were no significant differences in laparoscopic procedure between normalweight and overweight elderly patients [1-3]. There were no conversions to open procedure. Mean operative time was 116 minutes (range: 50 - 325). We analyzed preoperative and postoperative data not finding any significant difficulty difference in the laparoscopic approach in the elderly [10-13]. In primary inguinal hernias, in 37,5% of cases we used not absorbable polypropylene mesh, in 42,5% tridimensional polyester-collagen composite mesh and in 20% lightweight multifilament partly absorbable mesh.

Meshes were fixed in 82.5% of cases with absorbable fixation device, in 5% with not absorbable device and in 12.5% with fibrin glue.

In ventral hernias we used in all cases a polyester double-face mesh and fixed with absorbable devices. There was no difference as far as concerns the elderly quality of life between different meshes or fixation methods.

We had 6 major complications (14,24% - 2 chronic inguinal pain and 4 recurrences in inguinal hernias) [18,13] and 5 minor complications (11,90% - 3 seromas and 2 wound haematomas in ventral hernias) [11,7,9]. Mean hospital stay was 4,7 days (range: 1-18 days) in Group I and 1,2 days in Group II; all patients referred better quality of life after the procedure.

All procedures were performed by an expert surgeon or by a resident with an expert surgeon; there was no significant difference in intraoperative complications and operation time depending on the first operator.

## Conclusion

Laparoscopic repair of primary inguinal hernias and incisional ventral hernias with transabdominal placement of composite mesh in the elderly achieves excellent results with lower morbidity in comparison with open surgical approaches. In our experience, adequate fixation of the mesh, mesh extension to cover the entire defect and standardization of the placement space between the sutures are crucial to the success [10-12].

Despite the retrospective analysis of our sample, the study showed that elderly patients can be operated with laparoscopic approach with the same advantages that would be obtained if operated with open technique [13,14]. The resident with an expert surgeon can obtain the same results of an expert surgeon as far as concerns outcomes and morbidity.

We consider surgery approach more difficult in the elderly in some cases [15] but we also considered laparoscopic approach is, in general, a safe and feasible technique in acute pathology [16] and a safe approach also in the elderly [17,18]. In our experience, laparoscopic repaire of wound defects is a good standard technique also in the elderly.

## Competing interests

The authors declare that they have no competing interests.

## Authors' contributions

AGF: conception and design, interpretration of data, given final approval of the version to be published.

VM: critical revision, interpretation of data, given final approval of the version to be published.

SE: conception and design, interpretration of data, given final approval of the version to be published.

AF: acquisition of data, drafting the manuscript, given final approval of the version to be published.

SC: acquisition of data, drafting the manuscript, given the final approval of the version to be published.

EG: acquisition of data, drafting the manuscript, given the final approval of the version to be published.

AS: critical revision, interpretation of data, given final approval of the version to be published.

MS: acquisition of data, drafting the manuscript, given final approval of the version to be published.

## Declarations

Funding for this article came from personal funds.

This article has been published as part of *BMC Surgery *Volume 13 Supplement 2, 2013: Proceedings from the 26th National Congress of the Italian Society of Geriatric Surgery. The full contents of the supplement are available online at http://www.biomedcentral.com/bmcsurg/supplements/13/S2
